# Time–Frequency Mask-Aware Bidirectional LSTM: A Deep Learning Approach for Underwater Acoustic Signal Separation

**DOI:** 10.3390/s22155598

**Published:** 2022-07-26

**Authors:** Jie Chen, Chang Liu, Jiawu Xie, Jie An, Nan Huang

**Affiliations:** National Key Laboratory of Science and Technology on Communication, University of Electronic Science and Technology of China, Chengdu 610000, China; cliu.wcom@uestc.edu.cn (C.L.); 201622260214@std.uestc.edu.cn (J.X.); 202022220232@std.uestc.edu.cn (J.A.); 202121220218@std.uestc.edu.cn (N.H.)

**Keywords:** blind source separation, binary mask, deep learning, underwater acoustic signal

## Abstract

Underwater acoustic signal separation is a key technique for underwater communications. The existing methods are mostly model-based, and cannot accurately characterize the practical underwater acoustic communication environment. They are only suitable for binary signal separation and cannot handle multivariate signal separation. However, recurrent neural networks (RNNs) show a powerful ability to extract the features of temporal sequences. Inspired by this, in this paper, we present a data-driven approach for underwater acoustic signal separation using deep learning technology. We use a bidirectional long short-term memory (Bi-LSTM) approach to explore the features of a time–frequency (T-F) mask, and propose a T-F-mask-aware Bi-LSTM for signal separation. Taking advantage of the sparseness of the T-F image, the designed Bi-LSTM network is able to extract the discriminative features for separation, which further improves the separation performance. In particular, this method breaks through the limitations of the existing methods and not only achieves good results in multivariate separation but also effectively separates signals when they are mixed with 40 dB Gaussian noise signals. The experimental results show that this method can achieve a 97% guarantee ratio (PSR), and the average similarity coefficient of the multivariate signal separation is stable above 0.8 under high noise conditions. It should be noted that our model can only handle known signals such as test signals for calibration.

## 1. Introduction

At present, underwater acoustic communication [1] mainly uses sonar technology to detect, locate and identify underwater targets. However, sonar technology has to overcome effects from noise such as ship noise and ocean noise [2,3,4]. Therefore, a method of reducing the impact of noise is the most critical part of underwater communication. Source separation technology is a good method of reducing noise [5,6,7,8] that has attracted a great deal of attention from researchers in both academia and industry. Among these source separation methods, non-negative matrix factorization (NMF) [9] is one method that can be used to separate source signals. This method converts complex and sensitive feature extraction problems into non-negative matrix dimensionality reduction problems by extracting a set of basis vectors describing the underlying features of the target. However, the correlation of its features causes more similar-feature redundancy in the basis matrix and weakens the feature coefficients in the linear representation, which is not conducive to target recognition. Blind source separation (BSS) is also a classical method [10,11,12], consisting of a mathematical model, an objective function, a separation algorithm and evaluation criteria [13,14]. In research into the BSS algorithm, two approaches are always studied and employed. One is based on independent component analysis (ICA) [15], which works well when the number of sources *N* is less than or equal to the number of sensors *M*. The use of ICA is not limited to linear instantaneous mixing; it is also used to solve the separation problem for convolutional mixing and even nonlinear mixing. The other relies on the sparseness of source signals, which works well when *N* is greater than *M*, e.g., the binary T-F mask approach [16]. The binary T-F mask approach extracts a signal by calculating the binary masking matrix of the signal. It has the advantage of real-time operation, and in recent years it has also been applied to underwater acoustic separation in combination with underwater sound characteristics.

In view of the underdetermination in underwater acoustic communication, this study considers the binary time–frequency mask method based on sparsity. The traditional binary T-F mask method chooses features which are performed manually by using the observation signals. Due to the outliers and distribution of anisotropic variance, the traditional feature extraction method has certain limitations. It can only be used in binary signal separation, as the effect is poor in multiple signal separation and it cannot meet the requirements of separation accuracy. At present, the improvement of the binary T-F masking method remains a matter of feature design [17,18,19]. However, it is not easy for human experts to design good features. These artificial features are easily affected by outlier problems and have strict requirements regarding the selection of source location. As an alternative, in addition to traditional binary T-F masking, the method of extracting the original features of the underwater acoustic source using a deep neural network has shown good performance. This method has been used to solve image recognition, natural language processing (NLP) and even communication problems [20]. The deep learning approach [21,22,23] also represents a breakthrough in the separation of signals. Therefore, we extract the features of the underwater acoustic signals by means of a deep learning approach. The main contributions of this work are as follows:

(1) We propose a deep learning method based on Bi-LSTM. This method uses the powerful feature extraction capability of RNN and not only improves the performance in separating binary signals but also achieves good results in ternary or multivariate signal separation experiments. This overcomes the limitations of the previous separation of single targets from deep learning sources.
(2) We improved the training sample using the idea of embedding, i.e., embedding each T-F point into a high-dimensional space so that each T-F point can be represented as a vector, and then adding energy-based reference labels to the training sample. This makes the T-F points of different sources more distinct and makes clustering easier in the process of neural network learning.
(3) We carried out many experiments on the separation performance of this method by using unknown randomly generated noise and the marine noise actually collected. The experimental results show that with an increase in the number of clusters *K*, the effect of this method in separating noise improves further. It was proved that this method has the ability to reduce the noise impact of passive sonar platforms and to improve the recognition rate of underwater targets, which is significant for improving the performance of sonar positioning, detection and identification.

The rest of the paper is organized as follows. In Section 2, we introduce a traditional system model for underwater acoustic source separation. Then, in Section 3, we present a description of the proposed approach, including offline training and online testing. Section 4 presents the experiments. Finally, conclusions are drawn in Section 5.

## 2. Mainstream Method: Binary Time–Frequency Masking Method

The binary T-F mask approach separates the underwater acoustic signals according to the auditory masking, using the underwater acoustic source that dominates the energy in a certain T-F domain. Although the target signals received by the system have varying degrees of frequency-band overlap, the main energy of different target signals is usually hidden in different frequency bands. Hence, the binary mask approach can use this property to realize underwater acoustic signal separation by clustering the T-F bins. To cluster the T-F bins, the traditional method uses the observation signals and calculates manually to obtain the features.

### 2.1. Restrictions on Using Existing Methods

The use of binary T-F masking techniques must satisfy the sparsity condition. Since the sound signal is generally not sparse in the time domain, it must be transformed into the T-F domain by some transformation [24,25]. However, in the actual separation process of underwater acoustic signals, it is found that the energy of different underwater acoustic radiation signals is usually concentrated in different frequency bands, and the target radiation signals received by the system will show different frequency-band aliasing phenomena. The study found that as long as the underwater acoustic signal can satisfy the absolute dominant condition for the energy, the binary T-F masking algorithm can be used to achieve separation. This condition is written as:

(1) $$|X_{i}\left(t,f\right)|\gg |X_{j}\left(t,f\right)|,i\neq j,\forall t,f,$$

where $X_{i}\left(t,f\right)$ is the short-time Fourier transform (STFT) of signal $x_{i}\left(t\right)$. Using STFT, signals in the time domain can be transformed to the T-F domain, which can satisfy the property of sparsity. Geometric features for clustering are calculated based on this constraint.

This condition can also be understood as representing the fact that the overlap of the T-F domain is a relatively small portion of one of the underwater acoustic signals, so that ignoring the information in this part does not affect the recovery of the entire signal.

### 2.2. Signal Separation Steps in Underdetermined Case

**Figure 1:**
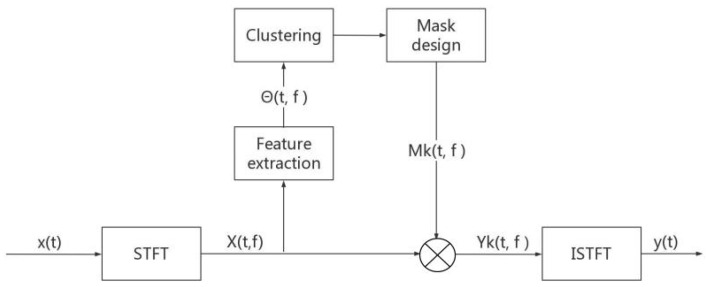
Example of T-F mask approach.

This approach is summarized in Figure 1. Based on the sparsity condition of absolute dominance of the energy, in the underdetermined case, the idea of using the binary T-F masking method for water acoustic blind separation is as follows:

(1) STFT. Let the sampling frequency of the observation signal be $f_{s}$ and convert the time domain signal x(t) into the T-F domain representation by using the T-point STFT transform:

(2) $$X\left(t,f\right)=\sum_{r=-T/2}^{T/2-1}x\left(r+tL\right)win\left(r\right)e^{-j2\pi fr},$$

where *t* is the time in seconds, *f* is the frequency in Hertz, *T* is the length of the window and *L* is the moving length of the window. Here, win(r) represents the window function. Commonly used functions are the rectangular window, Hanning window and Hamming window. In the subsequent inverse short-time Fourier transform (ISTFT), we used the Hanning window for the transformation, to ensure consistent parameters.

(2) Feature extraction. The source signal X(t,f) satisfying the sparse condition is obtained using the STFT transform, and the feature vector Θ(t,f) is calculated therefrom. In this eigenvector, there are differences between different sources which can be measured by distance. The eigenvector Θ(t,f) is generally composed of the geometric characteristic magnitude α(t,f) and the phase difference ϕ(t,f) between the observed signals. Taking two observation signals $X_{1}\left(t,f\right)$ $X_{2}\left(t,f\right)$ as an example, the eigenvector Θ(t,f), the order of magnitude α(t,f) and the phase difference ϕ(t,f) can be calculated using the following equations:

(3) $$\Theta \left(t,f\right)=\left[\alpha (t,f),\phi (t,f)\right],$$

(4) $$\alpha \left(t,f\right)=\frac{|X_{2}\left(t,f\right)|}{|X_{1}\left(t,f\right)|},$$

(5) $$\phi \left(t,f\right)=\arg\left(\frac{X_{2}\left(t,f\right)}{X_{1}\left(t,f\right)}\right).$$

The phase difference is usually normalized to avoid frequency sequencing problems, and the above equation can be written as:

(6) $$\phi \left(t,f\right)=\frac{1}{2\pi f}\arg\left(\frac{X_{2}\left(t,f\right)}{X_{1}\left(t,f\right)}\right).$$

Expanded to the case where there are multiple observation signals, the order of magnitude α(t,f) and the phase difference ϕ(t,f) are expressed as:

(7) $$\alpha \left(t,f\right)=\left[\frac{\left|X_{1}\left(t,f\right)\right|}{A(t,f)},\ldots \frac{\left|X_{n}\left(t,f\right)\right|}{A(t,f)}\right],$$

(8) $$A\left(t,f\right)=\sqrt{\sum_{j=1}^{n}{\left|X_{j}\left(t,f\right)\right|}^{2}},$$

(9) $$\phi \left(t,f\right)=\left[\frac{1}{\beta_{1}f}\arg\left(\frac{X_{1}\left(t,f\right)}{X_{B}\left(t,f\right)}\right),\ldots ,\frac{1}{\beta_{n}f}\arg\left(\frac{X_{n}\left(t,f\right)}{X_{B}\left(t,f\right)}\right)\right],$$

where, A(t,f) is the normalization coefficient of the order of magnitude; $\beta_{j}=\beta =4\pi d_{max}/c,j=1,\ldots ,n$ is the weight coefficient of the phase difference, subscript *B* represents the label of the reference observation signal, *c* represents the sound propagation speed and $d_{max}$ represents the maximum distance between the reference observation signal and other observation signals.

We express Θ(t,f) as a plural form with the following equation:

(10) $${\tilde{\Theta }}_{i}\left(t,f\right)=\left|X_{i}\left(t,f\right)\right|\exp\left[j\frac{\arg\left(X_{i}\left(t,f\right)/X_{B}\left(t,f\right)\right)}{\beta_{i}f}\right].$$

Normalization of the above equation yields a eigenvector representation of the observed multiple signal:

(11) $$\Theta_{i}\left(t,f\right)={\tilde{\Theta }}_{i}\left(t,f\right)/∥{\tilde{\Theta }}_{i}\left(t,f\right)∥,$$

(12) $$\Theta \left(t,f\right)={\left[\Theta_{1}\left(t,f\right),\ldots \Theta_{n}\left(t,f\right)\right]}^{T}.$$

From Equations (10)–(12), we know that Θ(t,f) is influenced by $\left|X_{i}\left(t,f\right)\right|$, $X_{B}\left(t,f\right)$, $\beta_{i}$ and *f*.

(3) Cluster analysis. Clustering the feature vector Θ(t,f) can result in m clusters $C_{1},\ldots ,C_{m}$ corresponding to m source signals. Past clustering methods include manual clustering [16], kernel density estimation [26] and the maximum likelihood (ML)-based gradient search method [27]. Because K-means clustering has the characteristic of simple, convenient and fast convergence, it has become the most commonly used method for cluster analysis. K-means can minimize the sum Y of the Euclidean distances (EDs) of each source signal and the corresponding cluster center $c_{k}$, and can automatically divide the samples into m clusters. The equation is expressed as:

(13) $$Y=\sum_{k=1}^{m}Y_{k},$$

(14) $$Y_{k}=\sum_{\Theta \left(t,f\right)\in C_{k}}{∥\Theta \left(t,f\right)-c_{k}∥}^{2}.$$

First, m cluster centers $c_{1},c_{2},\ldots ,c_{m}$ are randomly initialized, and each feature vector is assigned by iterating Equation (15). Then, the feature vector Θ(t,f) closest to the mean vector $c_{k}$ is found and assigned as a cluster:

(15) $$C_{k}=\left\{\Theta \left(t,f\right)|k=\underset{k}{\mathrm{argmin}}{∥\Theta \left(t,f\right)-c_{k}∥}^{2}\right\},$$

Then, we calculate the mean of all feature vectors belonging to $c_{k}$ and correct the cluster center:

(16) $$c_{k}\leftarrow E{\left[\Theta \left(t,f\right)\right]}_{\Theta \in C_{k}},$$

Substituting the updated mean vector into Equations (13) and (14) enables calculation of the objective function Y. If Y converges, then the set $C_{k},k=1,2,\ldots ,m$ corresponding to each source is obtained after the iteration ends.

(4) Binary T-F masking. Using the results obtained by clustering, a binary T-F masking matrix is constructed. The binary T-F masking matrix is a matrix consisting of values 0 and 1, whose size is consistent with the T-F matrix. This is similar to the binary test in spectrum sensing [28,29,30,31]. The matrix sets the mask value to 1 or 0 according to whether each T-F point belongs to the target signal, indicating whether the T-F-point information belongs to the source signal.

$$M_{k}\left(t,f\right)=\left\{\begin{array}{cc} 1, & \Theta \left(t,f\right)\in C_{k}\\ 0, & \mathrm{others} \end{array}\right.,$$

Substituting into the following equation gives the spectrum of the estimated signal:

(17) $$Y_{k}\left(t,f\right)=M_{k}\left(t,f\right)X\left(t,f\right).$$

(5) Inverse short-time Fourier transform (ISTFT). After obtaining the T-F domain estimation, the final step must complete the recovery of the time domain signal $y_{k}\left(t\right)$ using ISTFT and the overlap retention method [32]:

(18) $$y_{k}\left(t\right)=\frac{1}{A}\sum_{l=0}^{L-1}y_{k}^{d+l}\left(t\right),$$

When using ISTFT, the parameters must be the same as those of STFT using Equation (2). If A is a constant related to the window function, A=0.5T/L when using Hanning window, and $y_{k}^{d}\left(t\right)$ is expressed as follows:

$$y_{k}^{m}\left(t\right)=\left\{\begin{array}{cc} \sum_{f\in 0,\frac{1}{T}f_{s},\ldots ,\frac{T-1}{T}f_{s}} & Y_{k}\left(m,f\right)e^{j2\pi fr}\\ & mL\leq t\leq mL+T-1\\ 0 & \mathrm{others} \end{array}\right.,$$

where, r=t−mL.

### 2.3. Evaluation of Separation Performance

In order to verify the separation performance of the algorithm after adding noise, we simulated the binary time–frequency masking method. The T-F masking method requires the signal to meet the conditions of WDO or energy dominance. Therefore, the LFM signal was selected for the simulation to facilitate the aliasing operation of the signal for time and frequency. The detailed experimental process is described in Section 4. The experimental results show that when there is no noise, each signal can be recovered well, and the method can correctly divide the T-F region of each signal. Once noise is added, the performance deteriorates. The estimated masking matrix not only loses some of the information of the signal itself but also receives the T-F information of other signals.

## 3. Proposed Method

**Figure 2:**
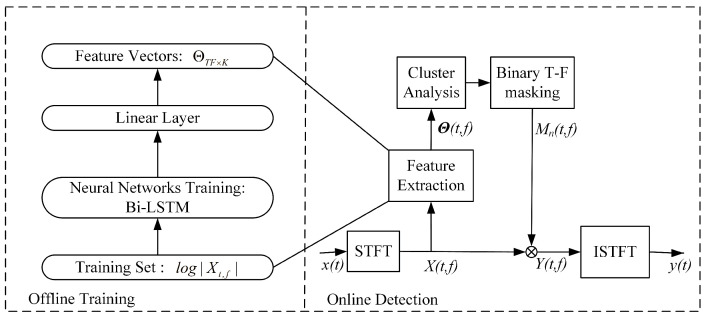
Framework of proposed approach.

In recent years, deep learning has been successfully applied in speech separation [33,34], and these previous attempts have generally assumed that the numbers and types of sources are fixed. However, in the case of underwater acoustic signal separation, we must consider two problems: (1) the model can be used to separate arbitrary types of underwater acoustic sources, i.e., the generalization problem and (2) the model can be used to separate arbitrary numbers of underwater acoustic sources, i.e., the scalability problem. Unlike previous attempts, in this article we use deep learning methods to learn a mapping for the input that is amenable to clustering, and this is helpful in overcoming the above two shortcomings. The architecture of the proposed method is illustrated in Figure 2.

**Figure 3:**
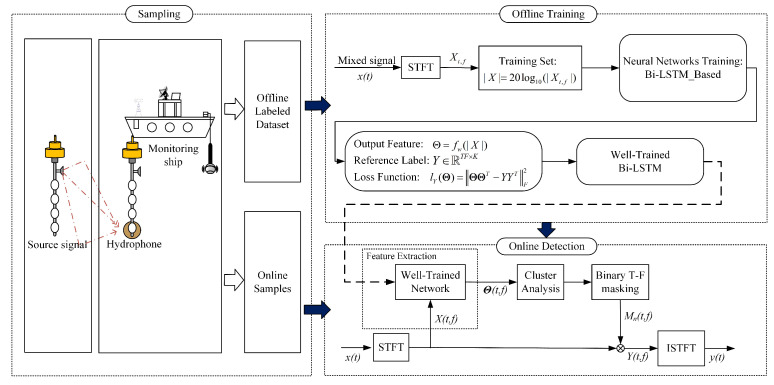
Flow chart of proposed method for underwater acoustic signal separation.

Based on the traditional binary T-F masking method, this scheme uses the deep neural network to extract features from the original underwater acoustic data instead of using artificial feature extraction. The program is divided into two stages: offline training and online testing. (1) Offline training phase. The training of the network consists of three parts: STFT, preprocessing and network training. The data are obtained from the measured underwater sound database and preprocessed to obtain training samples. Then, the T-F map of the underwater acoustic signal is obtained, mainly through STFT. Fianlly, it is sent to the network for training. In order to ensure that the network learns from the original underwater acoustic characteristics to obtain cluster-oriented features, this paper sets an appropriate objective function to make the characteristics of the network output easier to cluster. (2) Online testing phase. The artificial feature extraction method in the traditional binary T-F masking method is replaced by the network with the previous stage’s learning, and various mixed water acoustic signals are used to test whether the separation performance of the scheme meets the requirements. A flow chart for the specific method is shown in Figure 3.

### 3.1. Feature Extraction Based on Deep Neural Network

In order to achieve good separation performance after clustering, it is required that the clustering features have good distinguishing characteristics. In recent years, many studies have used deep neural networks [35] to obtain powerful characterizations for clustering [20,29,36,37,38,39,40,41,42]. Good results have been achieved in image recognition and NLP. These approaches are characterized by embedding the original data features into the new feature space, making the transformed features more suitable for clustering. In addition to the target underwater acoustic signal, ship-radiated noise and marine environment noise also exist in the sonar system. Due to varying degrees of decay in the ocean, the main energy of these types of noise is concentrated at different frequencies. The main sound-source frequencies are shown in Table 1.

For a communication sonar receiving transmitted signals from other sonar platforms, the receiving bandwidth of the receiver is about 100 Hz to 3000 Hz, and the receiver has prior knowledge of these detection signals [20]. According to the characteristics of underwater acoustic signals, a neural network can be used to “divide” different types of signals in the water audio frequency domain, using a Fourier transform signal processing method to restore the signal. Finally the target signal can be separated. According to the embedding principle, the role of the deep neural network used in this section is to map the original features (immediate frequency features) of the measured data to the new feature space. Each T-F point is converted into a vector. Each vector has a different position in the new feature space, depending on the amount of energy at the T-F point. These vectors are then “divided” into a number of reasonable ranges based on the distance between the vectors. That is, the T-F vectors belonging to the same underwater sound source have similarities, such that the distance is the smallest, and the T-F vectors belonging to different underwater sound sources have a large distance. Finally, they can be easily divided using a simple clustering algorithm.

Suppose a mixed water acoustic signal is transformed by STFT to obtain the original T-F characteristic $X_{t,f}\in R^{T\times F}$, where *t* is the number of time frames and *f* is the frequency point. Taking the logarithmic amplitude spectrum $20\log10(|X_{t,f}|)$ as the input of the network, for convenience of description, the latter is uniformly recorded as |X|. |X| can also be regarded as a sequence $\left[\chi_{1},\chi_{2},\ldots ,\chi_{T}\right]$ composed of spectral information $\chi_{i}\in R^{F}$ over a plurality of consecutive times. The deep neural network is parameterized by ω, and the features generated based on the network are expressed as:

(19) $$\Theta =f_{\omega }\left(|X|\right).$$

Here, $\Theta ={\left[\theta_{1},\theta_{2},\ldots ,\theta_{TF}\right]}^{T}\in R^{TF\times K}$ is the whole amplitude information |X| of the underwater acoustic signal, i.e., the cluster-oriented *K*-dimensional embedding feature learned by neural network. During the training process, the network sequentially maps the spectrum information $\chi_{i}$ to a new feature space at each time step and finally outputs it as an *F* × *K*-dimensional vector. This can be considered as encoding each T-F point in the original T-F feature $\chi_{i}$, and each T-F point after encoding is represented by a row vector $\theta_{j}$ of dimension *K*. Here $\theta_{i}$ is the unit vector, i.e., $|\theta_{j}{|}^{2}=1$.

The goal of training is to allow the line vector of the network output feature Θ to be divided into different water sources. That is, $\theta_{j}$ satisfies the vector distances belonging to the same water source, and the vectors belonging to different water sources are further away, thus achieving the purpose of separating the underwater sounds. Assuming that there is a mixed underwater sound in the water area, it is composed of C types of underwater sound sources:

(20) $$x\left(t\right)=\alpha_{1}s_{1}\left(t\right)+\alpha_{2}s_{2}\left(t\right)+\ldots +\alpha_{C}s_{C}\left(t\right).$$

Before sending the mixed signals to train the network, the energy of each source signal is compared at each time and frequency point. First, we set the reference label $Y\in R^{TF\times C}$ to divide the time and frequency points and compare the energy of these C types of underwater sound sources at various time and frequency points. The energy-dominated underwater sound source will mark the time and frequency points. For example, if the energy of the c-th (c∈{1,2,…,C}) underwater sound, dominates at the n-th (n∈{1,2,…,TF}) time and frequency points, then $y_{n,c}=1$. Therefore, the loss function of the model can be set as:

(21) $$\begin{array}{cc} l_{Y}\left(\Theta \right) & =∥\Theta \Theta^{T}-YY^{T}{∥}_{F}^{2}\\ & =\sum_{i,j}{\left(\langle \theta_{i},\theta_{j}\rangle -\langle y_{i},y_{j}\rangle \right)}^{2}\\ \; & =\sum_{i,j:y_{i}=y_{j}}\left(\right|\theta_{i}-\theta_{j}{|}^{2}-1)+\sum_{i,j:y_{i}\neq y_{j}}{\langle \theta_{i},\theta_{j}\rangle }^{2}, \end{array}$$

where ${∥•∥}_{F}^{2}$ is the squared Frobenius norm [43]. In the process of minimizing the loss function, two vectors for the same water source will become closer and closer, and the distance between two vectors for different water sources will increase. At the same time, since $\left(YP\right){\left(YP\right)}^{T}=YY^{T}$ exists for any permutation matrix P, the method can ensure that the label arrangement and the number of all training samples are independent.

### 3.2. Offline Training: Test Network Design Based on RNN, LSTM and Bi-LSTM, Respectively

Input and reference label processing: First, randomly take (2 *C*) underwater acoustic audio files from the file library and mix them according to Equation (20). Each audio file must be averaged before entering the network training stage:

(22) $$s^{\prime }\left(t\right)=s\left(t\right)-E\left[s\left(t\right)\right],$$

(23) $$s^{\prime \prime }\left(t\right)=\frac{s^{\prime }\left(t\right)}{max(|s^{\prime }\left(t\right)|)}.$$

The mixing coefficient α is randomly taken as an arbitrary number in the 16 s interval [3/4, 1]. According to Equation (2), the mixed signal has a window length of 32 ms and a time shift STFT of 8 ms, and the log amplitude spectrum X is taken. For a 16 s audio, it can be split into 500 samples of size 706. At the same time, we take the logarithmic amplitude spectrum of each source signal that makes up the mixed signal and compare the magnitude of the energy at each time and frequency point, to form the reference label Y with the same shape as X. To ensure local accuracy, each iteration consists of a sequence of time steps from multiple input samples of X and Y, and each sequence is 50% overlapped, to form a minimum batch-pair network for training.

In the offline training phase, in order to more clearly introduce the proposed Bi-LSTM structure used in this paper and highlight its superiority compared with other neural networks, we tested three structures: RNN, LSTM and Bi-LSTM. In addition, since LSTM is closely related to Bi-LSTM, the following section will first give a brief description of the LSTM structure, followed by a detailed introduction to Bi-LSTM.

Structure 1 (LSTM-based): RNN has long-term dependency problems. As the structural model of RNN becomes deeper, RNN must repeatedly apply the same operations to each moment in the long-term sequence to generate a very deep computational graph. Coupled with model parameter sharing, RNN is prone to losing the ability to learn previous information, making optimization extremely difficult. Unlike RNN’s regular loop body structure, LSTM uses neurons dedicated to memory storage. The neuron is a special network structure with three “gate” structures, called input gates, output gates and forgetting gates. During training, the LSTM relies on these gated operations (reset and read and write operations) to selectively influence the state of each moment in the network. After the investigation, we know that feature extraction can be performed using RNN. However, we use LSTM networks in this study, which is an improvement on RNN [44].

**Figure 4:**
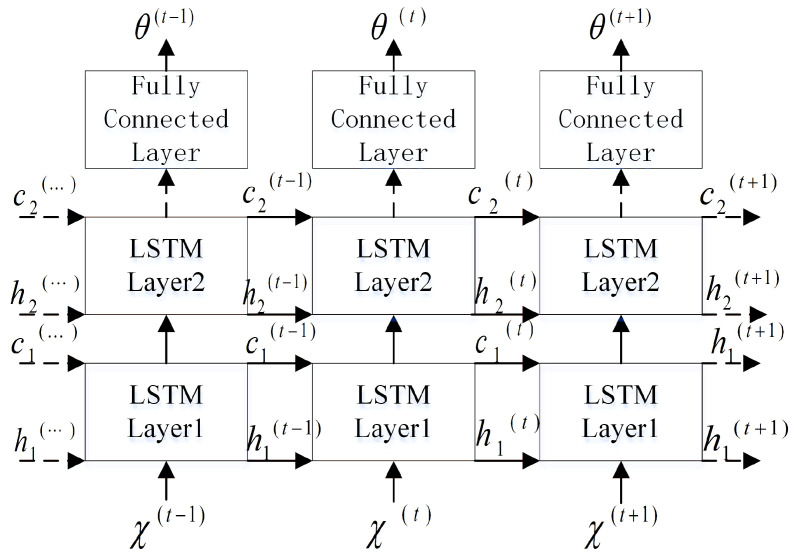
LSTM underwater acoustic separation network structure.

LSTM can form a deep LSTM network by stacking, repeating the loop body at each moment to enhance the expressive ability of the model. The parameters of the loop body of each layer are the same, and the loop body parameters of different levels can be different. A schematic diagram of the network structure for water acoustic separation using multilayer LSTM is shown in Figure 4. By stacking, the neural network can learn deeper expressions and finally embed them into the K-dimensional features.

Structure 2 (Bi-LSTM-based): The transmission of the two network structures, RNN and LSTM, is one-way from front to back, that is, the state at time *t* can only capture information from the past sequence $x^{1},\ldots ,x^{t-1}$ and the current input $x^{t}$. For some problems, however, the prediction of the output may depend on the entire sequence. For example, in speech recognition, some words currently have multiple interpretations and must be judged in context. Therefore, the processing of the voice must refer to the pronunciation information in the past and the future in order to produce a more accurate effect.

It is also possible to encounter the same problem in the field of underwater sound. For example, in underwater acoustic communication, sound waves are used instead of radio waves, due to the serious attenuation of underwater waves. Therefore, in underwater communication, the transmission of text, voice, images and other information needs to be converted into an electrical signal and then converted into an acoustic signal. At this time, in order to separate the speech signal in the water from noise such as waves, fish and ships, the influence of the front and back states on the output should be considered. During the collection of, and research into, marine sounds, the sound of fish as a signal for communication between fish schools should also consider the impact of the entire sequence on the output of the network. To this end, Bi-LSTM can be used to make full use of the context information in the sample for training.

**Figure 5:**
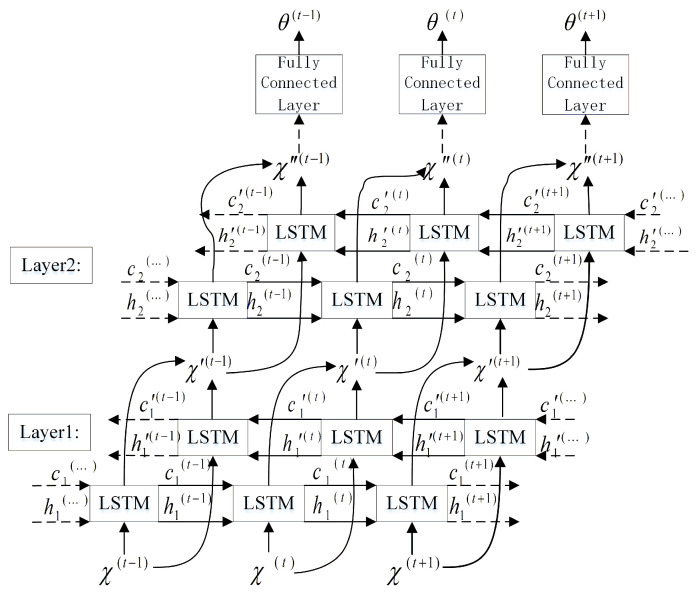
Bi-LSTM underwater acoustic separation network model diagram.

Bi-LSTM consists of two LSTMs of the same size and opposite starting points of the time series. Figure 5 shows the structure of a water acoustic separation network based on Bi-LSTM. Here, $h^{\left(t\right)}$ represents the state of the sub-LSTM that propagates information from t=1 to *T* (to the right) in time and ${h\prime }^{\left(t\right)}$ represents the state of the sub-LSTM in which the information moves backward from t=T to 1 (to the left) and can be obtained by substituting the reverse sequence into Equations (24)–(28). The specific operation of the unidirectional sub-LSTM layer is as follows. Given an input sequence $X=\{X_{1},\ldots ,X_{T}\}$, this model can be iteratively computed from t=1 to *T* and is composed of the following:

(24) $$i_{t}=\sigma \left(W_{Xi}X_{t}+W_{hi}h_{t-1}+W_{ci}c_{t-1}+b_{i}\right),$$

(25) $$f_{t}=\sigma \left(W_{Xf}X_{t}+W_{hf}h_{t-1}+W_{cf}c_{t-1}+b_{f}\right),$$

(26) $$c_{t}=f_{t}c_{t-1}+i_{t}tanh\left(W_{Xc}X_{t}+W_{hc}h_{t-1}+b_{c}\right),$$

(27) $$o_{t}=\sigma \left(W_{Xo}X_{t}+W_{ho}h_{t-1}+W_{co}c_{t}+b_{o}\right),$$

(28) $$h_{t}=o_{t}tanh\left(c_{t}\right),$$

where *W* and *b* are weights and biases, and *i*, *f*, *o* and *c* are the input gate, forget gate, output gate and cell activation vector, respectively. In addition, σ is the logistic sigmoid function. Therefore, at each time point t, the output unit can obtain information about the past sequence with respect to the input ${h\prime }^{\left(t\right)}$ and the relevant information about the future sequence of the input $h^{\left(t\right)}$. After the two sub-LSTM layers, we use a dense layer to obtain $\Theta_{t}$, which is the output of the $X_{i}$:

(29) $$\Theta_{t}=\phi \left(W_{h}h_{t}^{l}+b_{\Theta }\right),$$

where $h_{t}^{l}$ is the output of the final LSTM layer and ϕ is the ReLU activation function. By minimizing the loss value, some parameters will adaptively change as the learning process advances.

In the following experiments, we extracted the characteristics of the underwater acoustic signal using the above three networks (RNN, LSTM and Bi-LSTM) in the offline training phase. In the online test phase, combined with STFT and binary time–frequency masking methods, we obtained the corresponding experimental data for the three networks. The experiments showed that the Bi-LSTM structure had the best performance. However, the proposed scheme cannot handle the situation with unknown signal waveforms. It should be noted that our model can only handle known signals such as test signals for calibration; however, this is not an unreasonable constraint [45].

### 3.3. Online Test

Different models were trained and applied to the traditional binary T-F masking framework. The processing flow of the method is basically the same as the processing flow of the binary T-F masking method. The main steps are as follows:

(1) Select the underwater acoustic signal in the test set for mixing to obtain a mixed underwater acoustic signal. The signal is de-equalized and normalized, and the signal is subjected to STFT (the parameters of the STFT in the test phase are consistent with the STFT parameters in the training phase). Finally, |X| is obtained as an input.
(2) Using the trained model, the original feature X of the signal is transformed into a new embedded feature Θ. Since the new feature is just a matrix of dimension T×FK when it is output from the network, in the actual processing, we must reshape the data, convert their dimensions to TF×K, and facilitate the subsequent cluster analysis.
(3) Cluster analysis. The clustering analysis of feature Θ is performed using the K-means algorithm.
(4) T-F masking. According to the set $\Omega_{k}$ obtained by clustering, the corresponding binary T-F masking matrix $M_{k}\left(t,f\right)$ is set and substituted into Equation (17), thereby obtaining a T-F-domain estimate of the source signal.
(5) Time-domain recovery. The source signal ${\tilde{S}}_{k}\left(t,f\right)$ estimated in the above step is subjected to ISTFT estimation according to Equation (18), to obtain the time-domain waveform ${\tilde{S}}_{k}\left(t\right)$ of the source signal.

The clustering algorithm is used to classify this feature of the neural network output such that the vectors θ belonging to the same underwater sound source can be divided into a group. We set each “similar” vector to 1 and set the vectors that are not similar to 0. The new array dimension is reconstructed into a T×F matrix, which is the binary masking matrix corresponding to the water source.

## 4. Experiments

### 4.1. Experimental Conditions

For the experiments, we selected a hydroacoustic audio dataset in ShipsEar as a data sample [46]. Since its establishment, the database has been used for research on ship noise reduction, detection, identification, etc., especially for the application of deep learning technology [47,48,49]. The hydroacoustic data in this database were collected by the researcher David, a hydrologist from the Atlantic coast of northwestern Spain, and others from the University of Vigo in Spain. The composition of the database is shown in Table 2. The sonar audio, ship radiation noise and background noise form the A, B and C signals, respectively, and each audio file was selected to be about 6 seconds in length for testing. The sampling rate was unified to 44,100 Hz. In addition, we also simulated the binary time–frequency masking method. By comparing the effects of binary separation and multiple separation, the superior performance of the proposed method was proved. For the binary time–frequency masking method, we selected three LFM signals for simulation, which facilitates the aliasing operation of the signals for time and frequency. For the three LFM signals simulated, the sampling frequency was 50 kHz and the time length was 1 s. The specific parameters are shown in Table 3.

In the training stage, we attempted to train the model with a maximum mixture number of three. Hence, we randomly selected two or three files from the training set to mix in every iteration. Then, we used the model to separate each possible underwater acoustic mixing source. We designed the network structure with two LSTM layers with 600 hidden cells and a full connection layer with 100 cells, corresponding with the embedding dimension *K*. Stochastic gradient descent with momentum 0.9 and a fixed learning rate of $10^{-5}$ was used for training. The ReLU function was used as the activation function for the output layer, with order n. To prevent the network from overfitting and improve the generalization ability of the model, the input layer and the hidden layer’s dropout parameters were set to 0.2 and 0.5, respectively. When adding L2 regularization to the network, the parameter was set to $10^{-6}$. The number of training iterations of the model was 30.

In the test stage, the input feature *X* was the log magnitude spectrum of the mixed underwater acoustic signal, using STFT with 32 ms frame length, 8 ms window shift and the square root of the Hanning window. Moreover, the mixture was separated into 100 frames with half overlap to ensure the local accuracy of the output feature Θ. The masks were obtained by clustering the row vectors of the feature Θ. The number of clusters was set to the number of sources in the mixture.

### 4.2. Metrics

To evaluate the quality of the source separation, we used three quantitative criteria: (1) the preserved-signal ratio (PSR ∈[0,1]), representing the quality of the mask preserving the target source; (2) the signal-to-interference ratio (SIR ∈[0,∞)), representing the quality of the mask suppressing the interfering sources; and (3) the similarity coefficient ξ, which estimates the similarity between the signal $y_{i}\left(t\right)$ and the source signal $x_{j}\left(t\right)$.

PSR: The preserved-signal ratio (PSR) is used to measure the degree of protection of the masking matrix $M_{k}$ from the target signal $X_{k}\left(t,f\right)$. The mathematical equation is expressed as follows:

(30) $$PSR=\frac{∥M_{k}\left(t,f\right)X_{k}{\left(t,f\right)∥}^{2}}{∥X_{k}{\left(t,f\right)∥}^{2}},$$

The PSR characterizes the amount of energy remaining after the target signal passes through the masking matrix. In the equation, ${∥\cdot ∥}^{2}$ represents a double integral operation, that is, ${∥f(x,y)∥}^{2}=\;\int \int \;{\left|f(x,y)\right|}^{2}dxdy$. The PSR satisfies 0≤PSR≤1. If the estimated masking matrix $M_{k}$ satisfies the relationship ${\hat{M}}_{k}\subseteq M_{k}$ with the actual masking matrix ${\hat{M}}_{k}$, then PSR = 1.

SIR: The SIR indicates the suppression of the interference source by the masking matrix. An interference source composed of source signals other than the source signal $x_{k}\left(t\right)$ is denoted by $v_{k}\left(t\right)$, and the corresponding T-F domain is expressed as $V_{k}\left(x,y\right)$. The signal-to-interference ratio for the masking matrix M is defined as follows:

(31) $$SIR_{M}=\frac{∥M_{k}\left(t,f\right)X_{k}{\left(t,f\right)∥}^{2}}{∥M_{k}\left(t,f\right)V_{k}{\left(t,f\right)∥}^{2}},$$

where $SIR_{M}$ is a value greater than or equal to 0. The larger the value, the better the separation performance. When the masking matrix is completely suppressed with respect to the other source signals, $SIR_{M}=\infty$. In the T-F mask separation method, good separation performance requires that the T-F information of the source signal is preserved as much as possible and that the interference source can be suppressed, that is, the PSR is close to 1 and $SIR_{M}$ is as large as possible.

ξ: The similarity coefficient ξ is given by

(32) $$\xi_{ij}=\xi \left(y_{i},x_{j}\right)=\frac{\left|\sum_{t=1}^{n}y_{i}\left(t\right)x_{j}\left(t\right)\right|}{\sqrt{\sum_{t=1}^{n}y_{i}^{2}\left(t\right)\sum_{t=1}^{m}x_{j}^{2}\left(t\right)}}.$$

If $\xi_{ij}=1$, this means that the i-th estimated signal is exactly the same as the j-th source signal. If $\xi_{ij}=0$, this means that $y_{i}\left(t\right)$ and $s_{j}\left(t\right)$ are completely inconsistent. In an actual situation, due to the existence of the estimated difference, the separation performance of the similarity coefficient is generally close to 1, and the worst value is 0. Generally, these coefficients constitute a similarity coefficient matrix. If only one similarity coefficient in each row in the matrix tends to 1 and the others tend to 0, the separation performance is good.

### 4.3. Results

#### 4.3.1. Binary Signal Separation Using Binary Time–Frequency Masking Method

**Figure 6:**
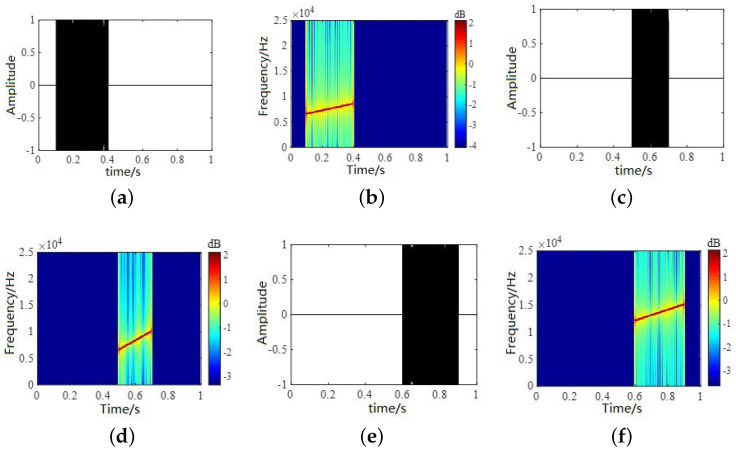
Time-domain waveforms and time–frequency diagrams for source signals. (**a**,**c**,**e**) show the time-domain waveforms of the three signals and (**b**,**d**,**f**) show the time–frequency diagrams for the three signals.

In the experiments, we first simulated three LFM signals that satisfy the energy-dominated condition. The time-domain waveforms and time–frequency diagrams of the simulation signals are shown in Figure 6. Figure 6a,c,e show the time-domain waveforms of the three signals, and Figure 6b,d,f show the time–frequency diagrams for the three signals.

**Figure 7:**
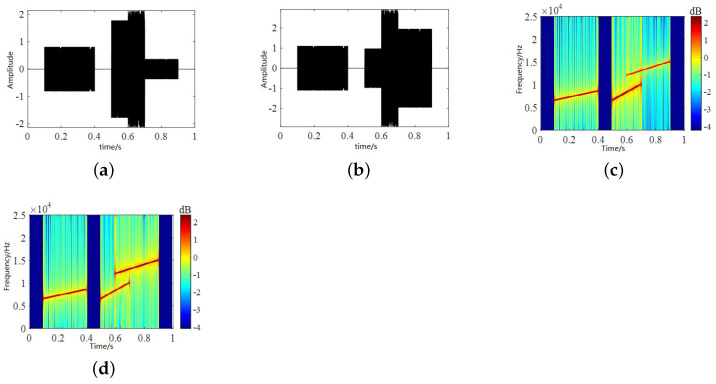
Time-domain waveforms and time–frequency diagrams of observed signals. (**a**,**b**) are the time-domain waveforms diagrams of the observed signals and (**c**,**d**) are the time-frequency diagrams.

**Figure 8:**
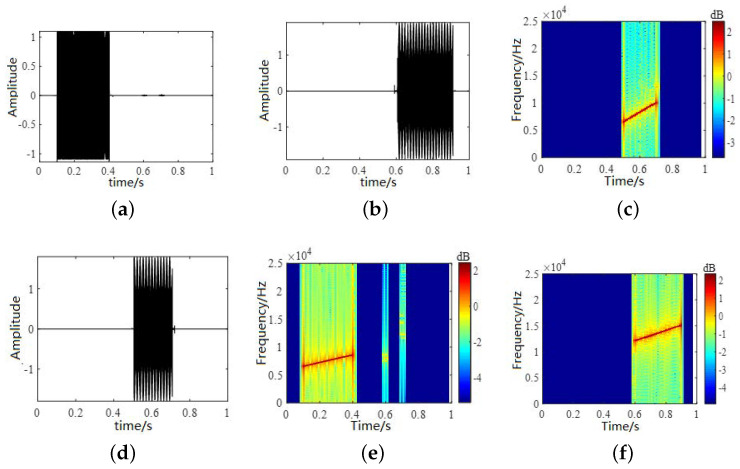
Time-domain waveforms and time–frequency diagrams of estimated signals. (**a**,**b**,**d**) show the time-domain waveforms of estimated signals and (**c**,**e**,**f**) show the time–frequency diagrams.

The randomly generated mixing matrix is linearly mixed according to Equation (33). In Equation (33), A represents the underwater channel matrix and n(t) represents white noise. The time-domain waveforms and time–frequency diagrams of the observed signals are shown in Figure 7. We selected the Hamming window for the observation signal, performed a 512-point STFT transformation and set a 25% overlap to obtain the time–frequency characteristics. According to Equations (7) and (9), we took the magnitude of the observation signal and the phase difference to form a feature vector, and finally obtained an estimated signal. The time-domain waveforms and time–frequency diagrams of the observed and estimated signals are shown in Figure 7. It can be seen from the results that when the signal meets the sparsity condition, the binary signal can be recovered using a binary time–frequency masking algorithm. The time-domain waveform and time–frequency diagram for the estimated signal are shown in Figure 8. From the effect diagram of the estimated signal, the source signal can be basically recovered using the binary time–frequency masking method. Source signal 2 is aliased with source signals 1 and 3 in the frequency domain and time domain, respectively, and so the information will be somewhat affected but can basically be recovered from the mixed signal.

(33) x(t)=As(t)+n(t).

The correlation coefficients ξ and the PSR and SIRM were measured under different signal-to-noise ratios. The results are shown in Table 4. It can be seen that when there is no noise, each signal can be recovered well. The two parameters PSR and SIR indicate that the method can correctly divide the time–frequency region of each signal, that is, the obtained masking matrix accurately covers the time of the signal and frequency information. Once noise is added, the performance deteriorates. The PSR reduction is small, but the SIRM reduction is obvious. This means that after adding noise, the estimated masking matrix not only loses some of the information of the signal itself but also receives time–frequency information from other signals.

#### 4.3.2. Binary and Multivariate Signal Separation Using the Proposed Method

**Figure 9:**
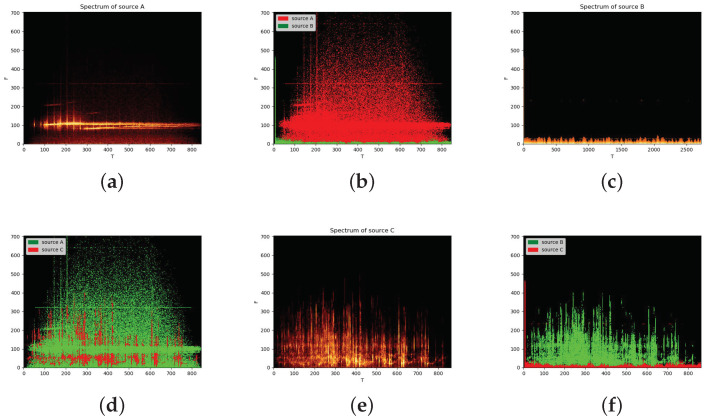
Visualization of separation of two-source mixtures. (**a**,**c**,**e**) show the spectrum of sources A, B and C separately and (**b**,**d**,**f**) show the separation results of the pairwise mixtures of A, B and C, respectively.

Next, we separated the mixed signals with two sources. The visualization of the result can be seen in Figure 9. We listed all possible combinations and observed the corresponding effect on separation. Figure 9a,c,e show the spectrum of sources A, B and C separately. Figure 9b,d,f show the separation results of the pairwise mixtures of A, B and C, respectively. Compared with the original spectra, it can be seen that the Bi-LSTM model can clearly separate the A, B and C signals before mixing.

In Table 5, we illustrate the de-mixing performance for separating two sources using the metrics mentioned in Equations (30) and (31). This shows that our proposed method had better performance in separating two sources, which indicates that this approach is different from many separation algorithms based on deep learning. SIR is infinity because the interfering sources are suppressed sufficiently, making the denominator close to 0 according to Equation (31).

**Figure 10:**
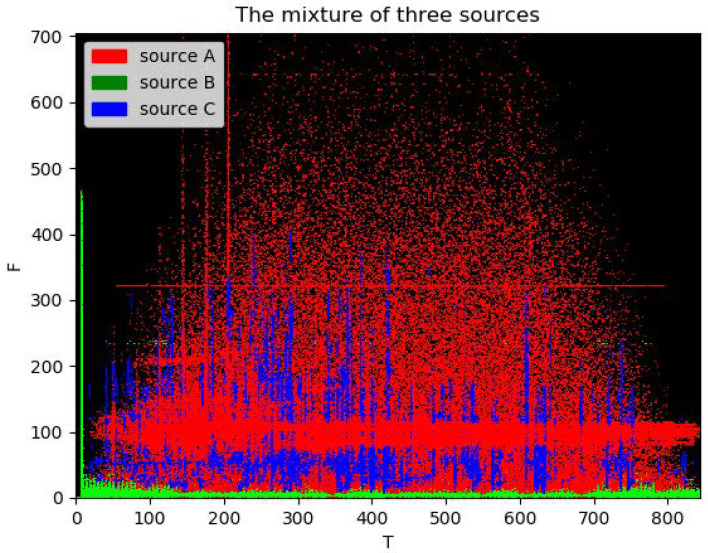
Separation results for three-source mixtures.

**Figure 11:**
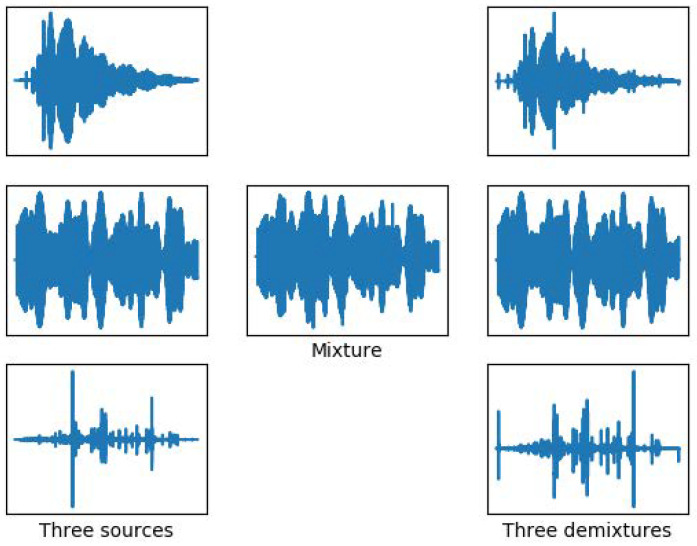
Three sources, the mixture and three de-mixed signals.

Furthermore, we separated mixed signals from three sources. Figure 10 and Figure 11 show an example of separating the mixtures of three sources. By comparing Figure 9 and Figure 10, it can be seen that the time and frequency points of each source can basically be found. The overlap between source signal C and source signal A is relatively large in the time–frequency domain. However, signal A dominates with respect to the energy at these overlapped time and frequency points, so it will not be disturbed by the signals and can basically be recovered. However, some information in signal C is lost. In fact, compared with background noise, people are more concerned about the loss of a sonar echo signal. Therefore, it is permissible to sacrifice part of signal C in practical applications. The overlap between signal B and signals A and C in the frequency domain is the least, and the separation performance is the best. However, in order to prove that using a deep learning method to separate underwater acoustic sources can achieve a breakthrough, we also show the results using the traditional binary T-F mask approach. In Table 6, the first example is our approach, and the second is the traditional approach. It is clear that our proposed method outperforms the traditional method, which cannot even separate sources C and A very well. What is more, compared with Table 5, when we separate more sources, the performance does not decrease too much. Therefore, the proposed model can be scaled up to more sources. Thus, it is appropriate for real-world applications when the number of sources is not fixed.

**Figure 12:**
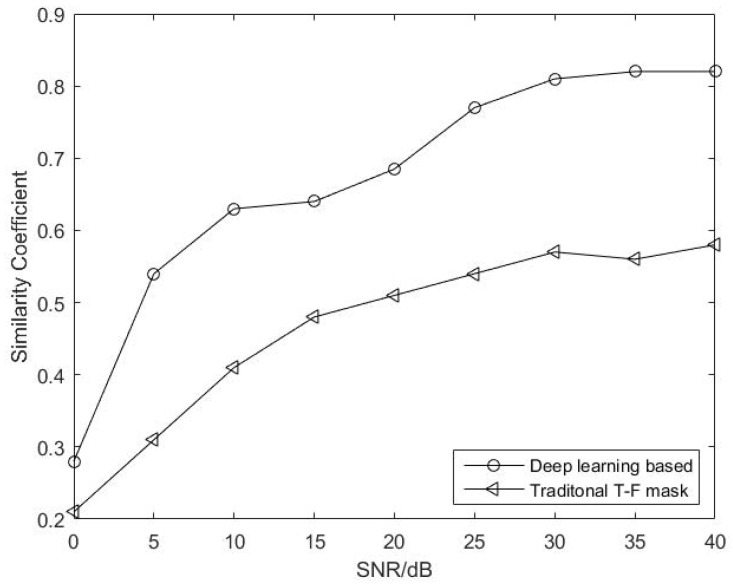
Comparison of similarity coefficients under different noise backgrounds.

In addition, considering that the mixed signal will be subject to interference from other unknown noises in the actual processing, Gaussian noise signals of 0–40 dB were added to the mixed signal, to analyze the separation performance under different SNR conditions. Meanwhile, compared with the traditional binary T-F masking method, the similarity coefficient was used as the measurement standard. The results are shown in Figure 12. When the noise background is relatively strong, both the deep-learning-based separation method and the traditional separation method will be greatly affected. As the noise is reduced, the estimated signal gradually becomes clear. Compared with the above separation situation, this test signal has larger aliasing in both the frequency domain and the time domain. Therefore, the traditional time–frequency masking method has a poor separation performance, and its final average similarity coefficient is stable at about 0.6. The deep-learning-based separation method can divide each target signal according to the energy-dominant condition, and therefore it has better separation performance on the whole.

**Figure 13:**
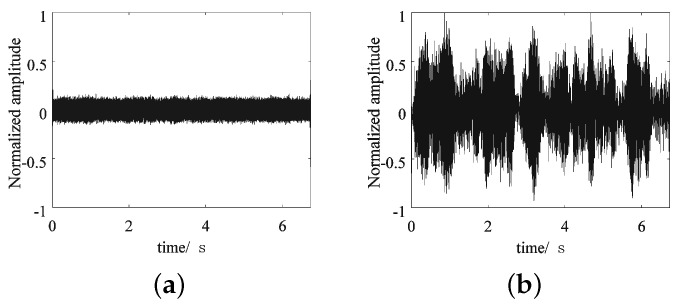
The separation results for signal C when different K values are set: (**a**) K = 3; (**b**) K = 4.

Under the condition of unknown noise, the separated signal will still carry noise, affecting the performance. It was found that the noise could be separated as long as the number of clusters was increased when the clustering algorithm was used. Taking the case of adding Gaussian white noise with an SNR of 0dB as an example, when the signal is divided into three categories, each signal will carry noise. Among them, signal C suffers the largest interference and has a very weak energy, as shown in Figure 13a. By increasing the number of clusters to four, that is, setting the K value of the K-means clustering algorithm to four, the proposed method could also separate noise from three source signals and recover the basic shape of signal C, as shown in Figure 13b. It can be proved that the proposed method can not only perform well in the separation of multivariate signals but can also work effectively in the presence of certain noise interference.

Finally, three models, RNN, LSTM and Bi-LSTM, were selected for comparison. Each model separated the mixed signals composed of sources A, B and C. The similarity coefficient ξ, and PSR and $SIR_{M}$ were selected as comparison indicators, and the comparison results are shown in Table 7.

According to the results of Table 7, RNN performed the worst. Although the PSR of the A and B signals reached 0.99, $SIR_{M}$ is very low. This shows that although the T-F information of the source signal was preserved, most of the T-F points that were not part of the source signal were also classified as source signals. The recovered signal then contains other signal components. In addition to retaining the original information well, LSTM and Bi-LSTM can also implement interference suppression for other signals. Bi-LSTM has a better suppression effect than LSTM. Signal B has the best recovery of the three configurations, especially in Bi-LSTM where the $SIR_{M}$ reached 10,576 and the PSR reached 0.97. Comparing the distribution of the three sources, it can be seen that signal B and signals A and C have almost no overlap in the frequency domain, and hence they are easily distinguished.

## 5. Conclusions

In this paper, a deep learning separation method for underwater acoustic signals based on the T-F mask method was proposed. The method mainly uses Bi-LSTM to create the features of the time–frequency mask for clustering. In this way, each T-F bin is “encoded” directly and partitioned into a reasonable region according to its magnitude. For real-world tasks, it is important for the proposed model to have good scalability, since the number of target sources is not fixed. At the same time, the model should have good generalization ability, so that it can work effectively when separating uncertain underwater acoustic mixed sources in online applications. In order to illustrate the universality and extensibility of the model, we conducted experiments on two unknown mixed sources and three mixed sources, respectively, and tested the robustness of the model by adding 0–40 dB Gaussian noise. Finally, we compared and analyzed the performances of the RNN, LSTM and Bi-LSTM networks in extracting underwater acoustic signal characteristics. The results showed that the proposed method could obtain better performance under the conditions of large mixed-signal uncertainty and large Gaussian noise, showing an obvious improvement compared with the traditional T-F mask method. The most important point is that compared with mainstream methods, this model not only has better separation performance for binary signal separation but can also effectively separate aliased signals in the case of multiple signal separation, which cannot be handled well by existing methods.

## Figures and Tables

**Table 1:** Major ocean sound-source frequencies.

Sound Source	Frequency
Marine life	0.5–20 KHz
Radiated noise from ships	less than 1 kHz
Surface ships	100–500 Hz
Submarines	100–500 Hz

**Table 2:** The composition of the database.

Category	Details
Number of recordings	90 segments
Recording length	15 s to 10 min
Number of ships	11
Background noise	Different depths and channel distances

**Table 3:** LFM signal parameters for binary time–frequency masking method simulation.

Signals	Frequency Range	Launch Time	Duration
LFM 1	6–8 kHz	0.1 s	0.3 s
LFM 2	6.5–10 kHz	0.5 s	0.2 s
LFM 3	12–15 kHz	0.6 s	0.3 s

**Table 4:** Separation performance at different SNRs.

SNR/dB	0	5	10	15	20	No Noise
*ξ*	0.60	0.62	0.74	0.81	0.89	0.98
PSR	0.71	0.72	0.82	0.85	0.90	0.98
$SIR_{M}$	5.82	5.47	15.56	27.93	316.21	24,193.72

**Table 5:** The de-mixing performance in the experiments.

Sources	SIR in (dB)	SIR out (dB)	SIR Gain (dB)	PSR
Source A	−14.28	∞	∞	0.93
Source B	14.28	∞	∞	0.92
Source C	−13.74	∞	∞	0.90

**Table 6:** Comparison of the de-mixing performance in Experiment 3 (top) with that of the conventional T-F mask approach (bottom).

Sources	SIR in (dB)	SIR out (dB)	SIR Gain (dB)	PSR
Source A	−14.29	∞	∞	0.94
Source B	14.10	∞	∞	0.93
Source C	−28.18	∞	∞	0.90
Source A	−14.29	13.93	28.22	0.81
Source B	14.10	∞	∞	0.93
Source C	−28.18	2.42	30.6	0.29

**Table 7:** Comparison of RNN, LSTM and Bi-LSTM models.

	A			B			C		
	*ξ*	*PSR*	*SIR_M_*	*ξ*	*PSR*	*SIR_M_*	*ξ*	*PSR*	*SIR_M_*
RNN	0.43	0.95	1.84	0.44	0.99	27.84	0.21	0.63	0.66
LSTM	0.91	0.96	91.99	0.82	0.94	205.88	0.71	0.73	8.74
Bi-LSTM	0.92	0.99	111.79	0.93	0.97	10576	0.77	0.76	8.68

## Data Availability

Not applicable.
